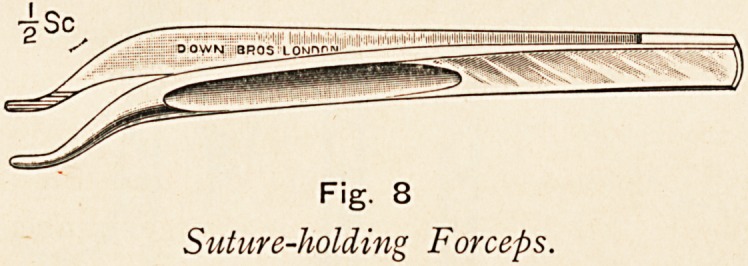# The Indications for Gastro-Enterostomy

**Published:** 1909-06

**Authors:** T. Carwardine

**Affiliations:** Surgeon to the Bristol Royal Infirmary


### THE INDICATIONS FOR GASTRO-ENTEROSTOMY.

BY

T. Carwardine, M.S.Lond., F.R.C.S.,

*Surgeon to the Bristol Royal Infirmary.*

---

We have been accustomed to regard chronic dyspeptics as, like the poor, always with us. The enrichments of modern surgery, however, enable us to restore many of them to health, by establishing an alternative route for the gastric contents, providing free exit in stasis, physiological rest when necessary for healing, and early neutralisation in hyperacidity.

In conjunction with various medical men, I have performed sixty or seventy operations for diseases of the stomach, and have formed some general impressions from them :—

1. An ulcer may present few or no symptoms, shown by old scars seen at operation, and perforations without antecedent history.

2. A dilated stomach in middle age, with a history of frequent indigestion years previously, indicates pyloric stenosis from old ulceration, especially when there is visible gastric peristalsis.

3. Gastric lavage should be abandoned. The necessity for habitual use of the stomach tube is sufficient indication for gastro-enterostomy.

4. Perforating ulcers are more commonly gastric.

5. Ulcers which give rise to mechanical digestive troubles are more commonly pyloric.

6. Ulcers which cause serious hemorrhage are frequently duodenal.

7. A history of hæmatemesis is rare in cases of gastric or duodenal ulcer which perforate.

8. Duodenal ulcers are far more common than hitherto recognised.

9. A healed gastric ulcer may not seriously affect a patient, unless it produce hour-glass contraction or adhesions to surrounding parts. On the other hand, healed ulcers in the pylorus or duodenum may seriously contract the lumen, and give rise to secondary gastric insufficiency.

10. Pyloric ulceration and stenosis in early life may arrest development, just as it affects nutrition in later life. Operation affords an impetus to development and nutrition, sometimes with temporary metabolic phenomena clinically, and sometimes with relief of nervous symptoms.

11. The risks of gastro-enterostomy, performed by an experienced surgeon on modern lines, are inconsiderable. It is now time that the remarkable advance in this direction should be fully realised. The risks for benign conditions should not be greater than those of the radical cure of hernia. The brothers Mayo record 300 successive cases with a mortality of under 1 per cent., and only 1 per cent. of secondary operations were required. I have personally done twenty-seven consecutive gastro-enterostomies without any leakage at all, and with only one death, due to old heart trouble and not to failure of technique.

12. "Vicious circle," following operation, should not be met with now—it is a sign of faulty technique. At most, a little transient bilious vomiting on the second or third day may exceptionally occur, probably due to œdematous swelling at the line of union.

Before deciding that gastro-enterostomy is indicated, it is important to determine whether it be contra-indicated, lest surgical aid should fail to alleviate or cure. Operation should not be done for mere pain and vomiting, symptoms common to many diseases. Nor should gastro-enterostomy be done, as a rule, unless the surgeon can confirm the diagnosis of some definite lesion at the time of inspection of the parts, though his ability to detect slight or less accessible lesions will improve with acquaintance. I cannot do better than quote the contra-indications given by my colleague, Dr. Nixon.[1]

**Contra-Indications :—**

1. Chronic alcoholism or drug taking.
2. Gastric crises.
3. Phthisis.
    (*a*) Dyspepsia.
    (*b*) Hæmoptysis.
    (*c*) Diaphragmatic pleurisy.
4. Mitral stenosis.
    (*a*) Pain.
    (*b*) Hæmoptysis.
5. Cirrhosis of liver.
6. Chronic Bright's disease (dyspepsia).
7. Renal colic.
8. Addison's disease (nausea, vomiting and faintness).
9. Overloaded or distended colon.
10. Arsenical poisoning.

To these might be added others, such as :—

11. Hysterical vomiting.
12. Gall-stone colic.
13. Dyspepsia of appendicitis.

There are certain **border-land cases** in which much care and judgement are necessary.

Unfettered by tradition, Dr. Nixon says, "Except during convalescence from an acute disease, I believe the diagnosis of 'atonic dilatation' should be received with the utmost scepticism. . . . Unless such a case yields very quickly to diet and treatment it should be explored. . . . As a disinterested observer of a very large number of surgical operations, I shall never make the

[1] *Bristol M.-Chir. J.*, 1908, xxvi, 224.

diagnosis of 'gastric neurosis' with complete assurance." On the other hand, we have the following observations from one of the most intrepid of surgeons: "We have but to look back on the discredit thrown upon surgery by the mutilating operations upon the pelvic organs of women to impel us to go slow on the numerous class of neurasthenic stomachs, and before we operate let us be sure the lesion exists elsewhere than in the mind of the patient" (Mayo).[1]

As far as my own personal experience goes, the most atonic and dilated stomachs I have handled have been those associated with prolonged pyloric stenosis. Next to them I would place the sagging stomachs of enteroptosis and slight degrees of pyloric contraction (? congenital), and the horse-shoe stomach which hangs like a bag of water with the cardiac and pyloric ends of the lesser curvature in contact. Perhaps the term "atonic dilatation of the stomach" in medicine may prove to have as little meaning as "atonic dilatation of the bladder" used to have in surgery. We now call the one urinary obstruction, and we are learning daily that the other may be the result of mechanical impediment more frequently than has been supposed.

**The indications for possible gastro-enterostomy may be :—**

1. Failure of reasonable medical treatment for benign conditions causing chronic dyspepsia.
2. All cases in which there is a suspicion of cancer. Surgery can only benefit these if they are explored early, before the "too late" signs of tumour and positive reactions to test-meals.
3. Chronic ulcer of duodenum or stomach.
4. Stenosis of duodenum or stomach (usually pyloric stenosis).
   - (*a*) Contracture from ulceration and scarring.
   - (*b*) Inflammatory tumour around ulcerations.
   - (*c*) Simple narrowing (? congenital).
   - (*d*) Hypertrophic pyloric stenosis of infants.
   - (*e*) From adhesions, *e.g.* to gall-bladder.
   - (*f*) Cancerous (under certain conditions).
5. Recurrent hemorrhage, duodenal or gastric.

[1] *St. Paul Medical Journal*, 1904, vi, 79.

6. Chronic dilatation of stomach, found at operation to be secondary to stenosis.
7. Hour-glass stomach.
8. Perforations in pyloric or duodenal regions (certain cases).
9. In gastrectomy (as part of the operation).
10. Duodenal fistula.
11. In marked gastroptosis—rarely.
12. *Acute dilatation of stomach* (?).

The success of gastro-enterostomy, and its low rate of mortality in the majority of cases of chronic dyspepsia which have not permanently yielded to medical treatment, justify the submission of such cases to the surgeon for his consideration, after excluding the contra-indicating causes of symptomatic pain and vomiting. It is not fair to the patient to withhold relief, and to permit the continued exposure to the greater risks of delay. The frequency with which **chronic dyspepsia** proves, on operation, to be due to some tangible cause, is a striking fact in modern practice.

The treatment of **cancer of the stomach** is on the eve of great advances. Its elusive tendency is great in the early stages, and its pathological vagaries are many ; but that cases are amenable to successful surgical relief, if only treated early enough, is beyond the shadow of a doubt. Therefore the time for operation is when there is a medical possibility of cancer, not a medical proof of the disease ; and there is no justification in waiting. The literature of the subject is overwhelming in indications that by the time a tumour can be felt clinically, and test-meals give somewhat definite (though often uncertain) results, the condition is too advanced for much hope from surgery. Gastro-enterostomy affords little advantage in advanced cancer of the stomach, when the mortality is as great as that of radical excision in earlier cases, and the relief granted to patients is so temporary that the operation is not in general favour with surgeons, except, perhaps, as a measure preliminary to gastrectomy, although most surgeons of experience prefer to perform the complete operation at one sitting. Sixty per cent. of cases of cancer of the stomach occur at the pyloric end ; the fundus remains free from involvement for a long time; and wasting, vomiting and distaste for food in a patient past middle life should excite suspicion. A diminution of HCl appears to be the only clinical test to which any real value can be attached, but it is not to be depended upon, especially when cancer supervenes upon chronic ulcer. Out of 1,200 cases, 400 came to operation, and the chemical and microscopical examination of the contents proved of little value. (Mayo.)

In **chronic ulceration of the stomach and duodenum,** and in its complications and sequelæ, gastro-enterostomy yields brilliant results, and to the pathological study of which modern surgery has contributed so largely. The stomach and duodenum should be considered as separate entities no longer. Physiologically they are complements one to the other; embryologically, the fore part of the alimentary canal ends below the bile-papilla, where the digestive portion of the gut ends; and Ochsner has shown that a muscular sphincter surrounds the duodenum an inch below the opening of the biliary and pancreatic ducts. The introduction of the pyloric sphincter merely restrains the gastric contents till prepared for duodenal digestion. It is a strange fact that cancer is so common in the stomach and so rare in the duodenum; also that simple ulceration is common to both, yet so rarely diagnosed as duodenal. Gastric ulcer is far more common in women, and duodenal ulcer more common in men; but surgical experience indicates that the proportion of duodenal ulcers generally is much larger than has been supposed hitherto. Out of 200 cases operated upon by the Mayo brothers, 87 had ulcers in the stomach, 98 in the duodenum, and 15 in both; although their earlier experience (1904) gave a much lower percentage of duodenal ulcers. My own experience bears out the greater proportion of duodenal ulcers seen at the time of operation, and causing symptoms apart from perforation, viz. 10 duodenal to 8 gastric, with ulcers in both duodenum and stomach in 3. Duodenal ulcers appear to be the more common amongst the cases coming to the surgeon for gastro-enterostomy, and for that and other reasons the effects of duodenal ulceration may be looked upon as more serious than those of gastric ulceration.

What is the ordinary outlook for cases of chronic ulcer ? If the 500 cases of "gastric" ulcer at the London Hospital (1897–1902) be taken as a guide[1] we find that—

10 per cent. died from perforation and peritonitis.
2.5 per cent. died from hæmatemesis.
5.5 per cent. died from other causes.
42 per cent. relapsed.

*i.e.* 60 per cent. died or were not properly cured. Without taking these statistics too seriously, we may ask for the general experience of physicians, as to what proportion of their patients with chronic ulcer have eventually become quite well and able to pursue an arduous life afterwards ? Have 10 to 25 per cent. of them died directly or indirectly from the lesion, as stated by many observers ? What proportion of enfeeblement ultimately follows in those who survive ? How many develop cancer later on ?

An unhealed ulcer of the stomach in later life must be regarded with suspicion as a precancerous condition, a large proportion of cancers occurring by changes in previous ulcers, and at the site of election for ulcers, viz. the pylorus and adjacent lesser curvature of the stomach. In 180 cases in which part of the stomach was resected, cancer on ulcer base was demonstrated in 54 per cent. (Mayo). The frequency of cancerous change in gastric ulcer is at least 50 per cent. (Rodman), and estimates of the previous history of ulceration in cancer cases have varied between 14 and 90 per cent.

In **stenosis,** commonly pyloric, and most frequently due to contraction from ulceration, the effects of gastro-enterostomy are perhaps the most marked. Such cases are associated with **dilatation of the stomach** in variable degree ; in some the viscus is atonic and thin, like a flaccid bag, hanging down as low as the pubes ; in others there are indications of muscular hypertrophy, which may be very marked towards the pylorus. Visible peristalsis may be present, succussion splash constant, gastrectasis and gastroptosis demonstrable on distension of the stomach,

[1] *Brit. M. J.*, 1902, ii, 1650.

and the condition verified by a bismuth meal with skiagraphy. The vomit is in large quantities at intervals, and of acrid odour, containing undigested food and perhaps sarcinæ. The patient is thin and inactive, the pulse soft and small, and occasionally there are symptoms of gastric tetany. The pyloric ulceration may be multiple, and in such cases, in particular, an inflammatory tumour forms, which is very frequently regarded as malignant. The results of gastro-enterostomy are pronounced and almost immediate, and the haggard wreck soon becomes a plump and industrious individual. In hypertrophic pyloric stenosis of

Fig. 1.
*Pyloric ulceration, stenosis and dilatation (benign).*

Fig. 2.
*Pyloric tumour, ulceration; muscular hypertrophy (benign).*

infants, and in partial degrees of congenital stenosis met with in adults, the operation may be employed.

In **recurrent hemorrhage** the indications for gastro-enterostomy are pronounced, for most of those acquainted with the subject agree that no attempt should be made to find the bleeding-point as a rule. I believe such cases are more frequently of duodenal origin. A duodenal origin was determined in five out of six cases for which I have been asked to operate, although hæmatemesis, as well as melæna, had occurred in the majority; and the operation was of lasting benefit. Duodenal ulcer is four times as common in men as in women, and most of my patients with hemorrhage were males in middle life. Duodenal ulcer has been found in the new-born, and at the age of 94.

**Hour-glass stomach**, of different grades, is now frequently met with in operations for benign diseases of that viscus, and is usually due to contracture following old ulceration. The proportion I have found to be about 15 per cent., and in two cases stenosis at the constriction was sufficient to demand plastic operation. Marked constriction is an indication for gastro-jejunostomy, the anastomosis being made with the proximal pouch ; and at the same time a plastic operation or anastomosis

Fig. 3.

*Duodenal ulceration with severe hemorrhage.*

Fig. 4.

*Duodenal ulceration, tumour; gastric dilatation.*

should be employed between the proximal and distal pouches. Most cases of hour-glass stomach are not diagnosed as such before operation, though the condition may be suspected, and certain tests applied in confirmation.

In **perforations** the indication for gastro-enterostomy is contraction of the pyloric orifice, the perforation being situated in that region. Opinions are somewhat divided as to the desirability of gastro-enterostomy at the time of closure of the perforation, or of leaving it till later. I have performed the simultaneous operation on one patient with recovery, and in another case which recovered after perforation I advised the patient to come up later for gastro-enterostomy, owing to the amount of pyloric stenosis. In subacute perforations, when old and firm adhesions to the liver have resulted, primary gastro-enterostomy may be resorted to without disturbing the adhesions.

In **gastrectomy,** for malignant or complicated benign disease, the operation is completed by lateral anastomosis of the dome of the stomach with the small intestine, a safer procedure than end-to-end union with the duodenum.

With marked **gastroptosis,** associated with enteroptosis or

Fig. 5.
*Hour-glass stomach. Duodenal ulcer.*

Fig. 6.
*Perforated gastric ulcer; gastro-jejunostomy.*

nephroptosis, the sagging of the stomach may be so great as to cause symptoms, and contribute to real distress. Such cases have to be carefully weighed, and in one patient, the daughter of a medical man, I fixed both kidneys and performed gastro-enterostomy, much to the patient's benefit. In this case there was a slight degree of simple narrowing of the pylorus (? congenital), and the main symptoms were vomiting and prostration, both being relieved by operation.

Gastro-enterostomy has been employed for **duodenal fistula** in combination with pyloric exclusion, and has been suggested for **gastric tetany.** It has been proposed for acute dilatation of the stomach, but its employment for this condition is open to criticism.

With regard to the details of the operation for gastro-enterostomy, surgeons of most experience in the operation habitually use clamps for the stomach and small intestine. Some years ago I designed twin stomach forceps, similar to my

Fig. 7.
*Author's Gastro-enterostomy Clamp.*

intestinal clamps. They are referred to in the latest edition of Jacobson's *Surgery*, but I refrained from previous personal publication of them until satisfied with their use. It should be noted that they are employed without any covering of rubber tubing, and I have not met with any technical defect in their use, or damage to viscera.

In the application of the continuous sutures uniting the stomach and small bowel, I have found a specially designed forceps very useful indeed. The assistant holds them in the *palm* of the hand, and grips each suture as it is passed, close down, to maintain tension on the last stitch as the next is being passed. The forceps being held horizontally, his hand is out of the way, and the tapering points enable any loops of suture to pass readily over them. The springs are much weaker than those of ordinary dissecting forceps, so that they do not tire the hand. After long experience of their use I venture to make them known as useful aids in gastric and intestinal anastomosis.

Some discussion has recently taken place as to the best line of anastomosis, whether anti- or iso-peristaltic. The chief point seems to be to avoid any twist of the bowel round its axis. I

Fig. 8
*Suture-holding Forceps.*

perform the anti-peristaltic operation of Mayo, and, like him, apply the forceps with the handles towards the operator standing on the right side. Those who prefer the iso-peristaltic, or vertical disposition of the bowel, apply the forceps with the handles towards the assistant. The experiences of the one group are quite the opposite to those of the other group, and I think the application of the forceps, from one side or the other, explains the difference. Personally I employ my forceps in the same way as Mayo employs Doyen's forceps, and make an antiperistaltic union with similar satisfactory results, and see no reason to revert to iso-peristaltic, as Moynihan (who applies forceps differently) has done.

The advantages of removing mucous membrane were first brought to the notice of the profession by Littlewood,[1] and I have adopted this procedure with various modifications.

[1] *Lancet*, 1901, i, 1817.

## Figures and Tables

**Fig. 1. f1:**
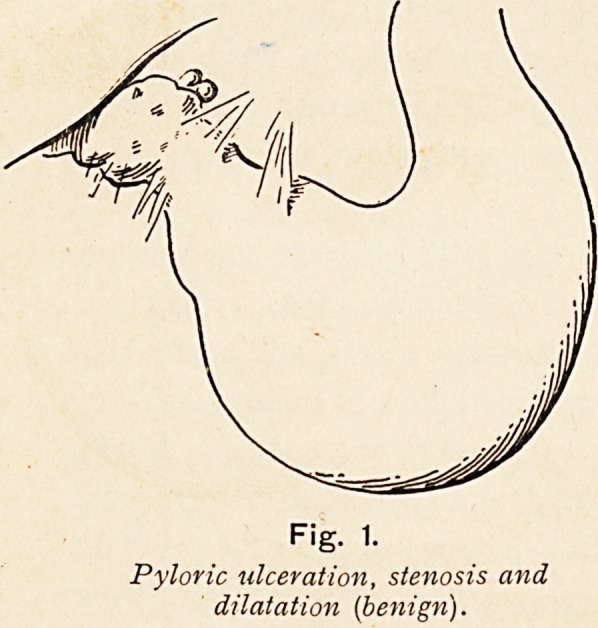


**Fig. 2. f2:**
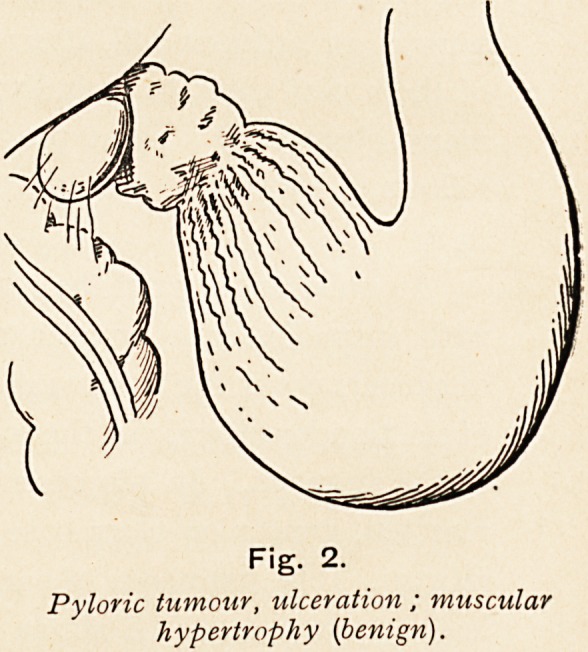


**Fig. 3. f3:**
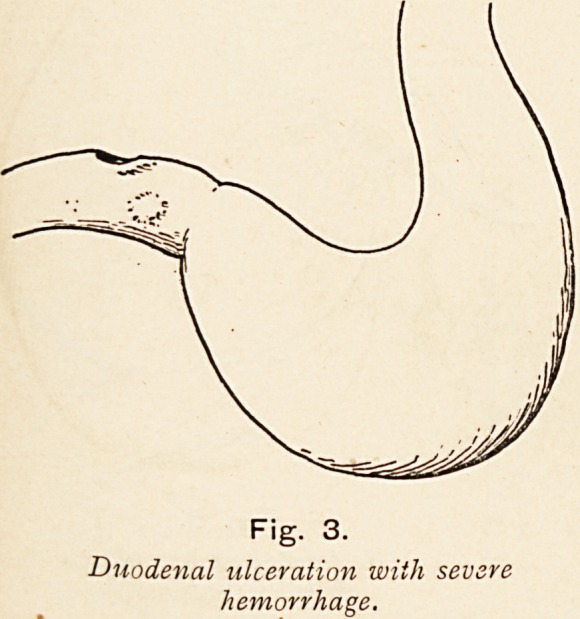


**Fig. 4. f4:**
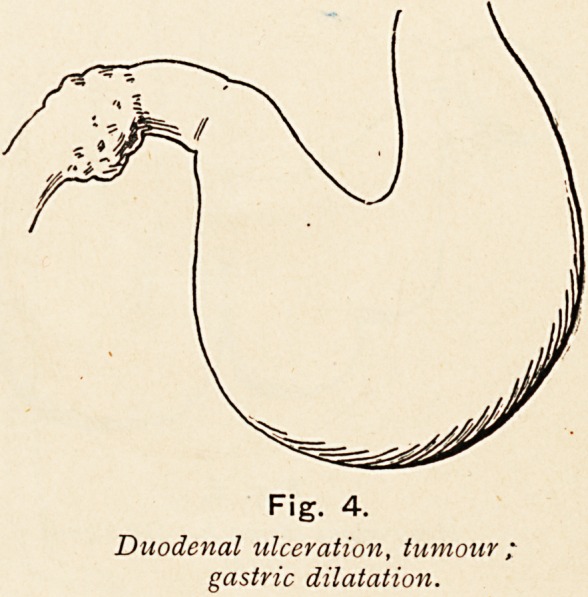


**Fig. 5. f5:**
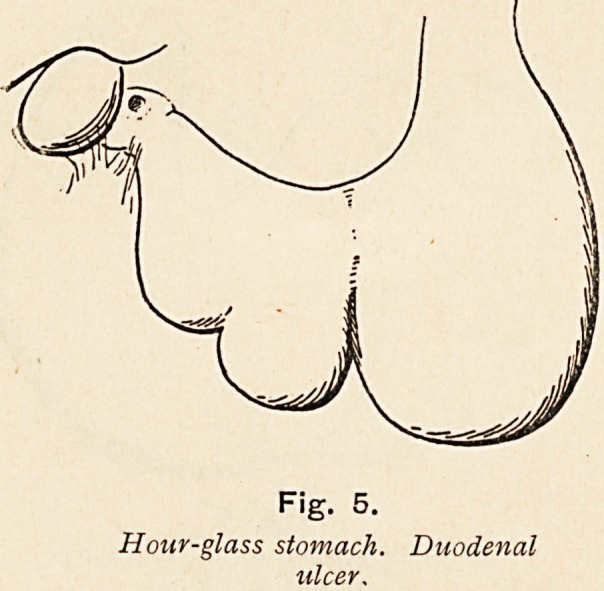


**Fig. 6. f6:**
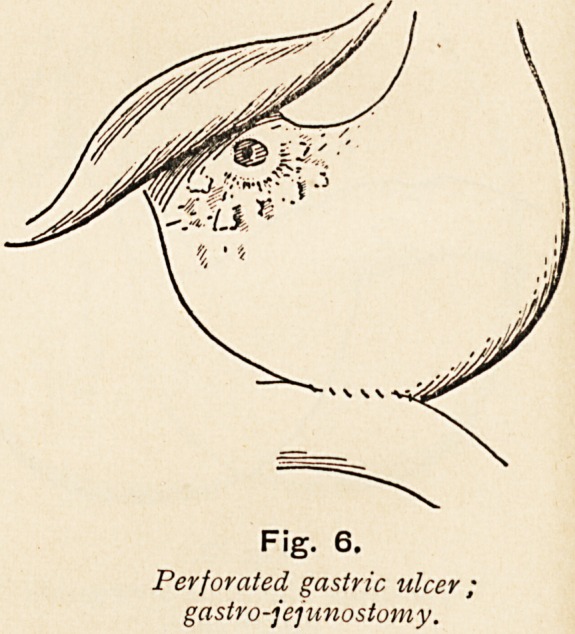


**Fig. 7. f7:**
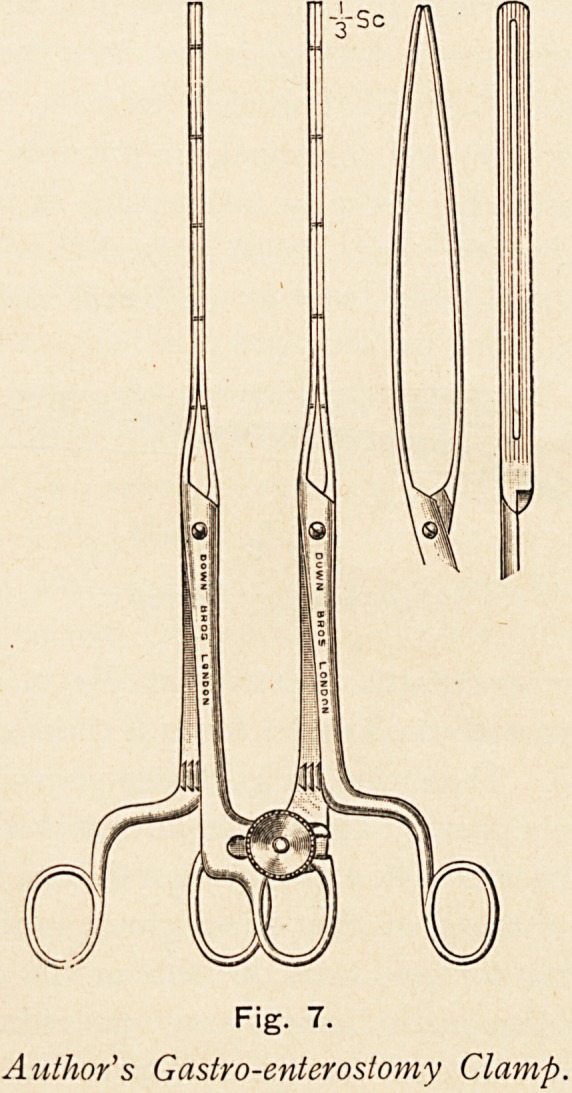


**Fig. 8 f8:**